# Principal Component and Structural Element Analysis Provide Insights into the Evolutionary Divergence of Conotoxins

**DOI:** 10.3390/biology12010020

**Published:** 2022-12-22

**Authors:** Akira Kio V. Kikuchi, Lemmuel L. Tayo

**Affiliations:** 1 School of Chemical, Biological, and Materials Engineering and Sciences, Mapúa University, Manila 1002, Philippines; 2 School of Health Sciences, Mapúa University, Makati City 1200, Philippines

**Keywords:** conus, conotoxin, divergence, molecular docking, prey shift, principal component analysis, structural element analysis

## Abstract

**Simple Summary:**

Conotoxins are small, structured components found in the venom of predatory cone snails. They were proven to be valuable probes and models for drug discovery and protein evolution studies. Conotoxins present an opportunity to study protein divergence and discover potential human therapeutic landscapes. Although there is considerable literature on conotoxin evolution and activity, what pushed conotoxin divergence remains unclear. Hence, in this paper, we conducted a two-phase study that investigated conotoxin evolution in terms of divergence, followed by structural analysis to determine the relevant structural elements. By understanding the evolution of conotoxins, we identified patterns that account for their superior specificity. The results revealed similarities based on the cone snail’s diet preference. The structural elements are in synch with their target prey preference as if cone snails evolved to fine-tune their conopeptide armory to respond to evolutionary pressures by producing conotoxins selective for their prey of choice. We identified several structural elements that account for this specificity. Conservation patterns are observed within diet subgroups but are divergent from other groups.

**Abstract:**

Predatory cone snails (*Conus*) developed a sophisticated neuropharmacological mechanism to capture prey, escape against other predators, and deter competitors. Their venom’s remarkable specificity for various ion channels and receptors is an evolutionary feat attributable to the venom’s variety of peptide components (conotoxins). However, what caused conotoxin divergence remains unclear and may be related to the role of prey shift. Principal component analysis revealed clustering events within diet subgroups indicating peptide sequence similarity patterns based on the prey they subdue. Molecular analyses using multiple sequence alignment and structural element analysis were conducted to observe the events at the molecular level that caused the subgrouping. Three distinct subgroups were identified. Results showed homologous regions and conserved residues within diet subgroups but divergent between other groups. We specified that these structural elements caused subgrouping in alpha conotoxins that may play a role in function specificity. In each diet subgroup, amino acid character, length of intervening amino acids between cysteine residues, and polypeptide length influenced subgrouping. This study provides molecular insights into the role of prey shift, specifically diet preference, in conotoxin divergence.

## 1. Introduction

Conotoxins are hyper-diverse short peptides isolated from predatory cone snail (*Conus*) venom. They are produced in their venom ducts and used as a part of an active hunting mechanism [1,2]. Over the years, conotoxins have been of interest to modern biology due to their intriguing diversity and applications [3,4,5,6,7,8]. Conotoxin evolution in terms of diversity is an attractive model for understanding the biology and evolution of adaptive traits and protein functions [9]. Understanding how peptide diversity arises in venom can aid in building models for elucidating ligand binding modes, understanding protein functions, discovering products, and permitting the reconstruction of multiple facets of the evolutionary trajectory by assembling manipulatable systems to directly test critical evolutionary questions [8,10,11].

Transcriptomic and proteomic studies revealed a compelling positioning of conserved and hypervariable regions within the same translational product. It is believed to play a role in maintaining structural stability and specificity while increasing divergence [1,12]. Several studies reported that diet preference is a major force driving venom composition patterns and diversity, and it is closely related to the species’ ability to capture and apprehend prey [13]. Although diet is a dominant force governing diversity across disparate venomous taxa, there are cases where diet preferences do not attribute to venom composition, and these claims challenge the generalizability of the pattern [13,14]. Hence, it is unclear whether a relationship between dietary preference and venom diversity must be expected, and these observations must be tested in a phylogenetically controlled and wide-scale setup. 

Until now, novel conopeptide sequencing efforts revealed the increasing diversity of peptide sequences. This diversity is presumed to be crucial in maintaining structural integrity and specificity to target receptors. With this remarkable diversity of conotoxins, cone snail venom presents an opportunity for studying the divergence of large gene families. Although there is considerable literature on conotoxin evolution, a broad genus analysis has seldom been documented. Most studies have only focused on one superfamily [15] and specific isolates in a cone snail species [16]. Not much has been conducted to analyze a large set of conotoxin data, such as a wide genus analysis, because such wide-scale investigation from sequencing to phylogenetic analysis can be tedious and expensive. However, including big data information in understanding divergence is vital for uncovering possibly valuable clues about conotoxin evolution. Investigating patterns with a large dataset may provide insights on the intermediate steps, patterns, and directions of conotoxin evolution in terms of function diversity. 

A practical approach for handling large peptide datasets involves bioinformatics, particularly multiple sequence alignment. The alignment behaviors of short peptides provide insights into evolutionary processes that have dispersed across multiple families [17]. Multiple sequence alignment (MSA) quantifies amino acid similarities and patterns in protein families. MSA is a critical tool for generating and testing hypotheses based on peptide structures, sequences, and functions [18]. However, computational limitations, time requirements, and accuracy limit MSA algorithms [19]. For every successful MSA run, assigning importance to specific amino acid positions in large protein families is challenging as differences among samples consist of multiple dimensions and are difficult to interpret. Usually, relationships between sequences are summarized in dendrograms. However, in handling large protein sequences, this method poses challenges to data analyses, ending in non-inferable data relationships [20]. 

Statistical methods are some of the many reported methods explored to solve the problem. They often involve numerical conversions to estimate the relationships quantitatively [17,21]. Fast Fourier transforms (FFT) and principal component analysis (PCA) have been applied to study large protein sequence alignments [22] and whole genomes [20]. Katoh et al. introduced a novel method for multiple sequence alignment based on FFT [22]. It allowed for rapid detection of homologous segments, improved the scoring system, and drastically reduced CPU time. They developed a program package called MAFFT (for multiple alignment using fast Fourier transforms), which incorporates new techniques described in their study [22]. As a multivariate analysis, PCA is a powerful dimensional reduction technique that shows advantages in data simplification. PCA for protein sequence analysis was first introduced by Casari et al. after representing amino acid residues as vectors in a generalized sequence space to project these vectors onto a lower-dimensional space [23]. Gogos et al. then used PCA to annotate genome sequences to sort new homologs to their respective subfamilies [24]. Recently, PCA has been applied to study protein sequence alignment and whole genomes to reduce and simplify the complexity of MSA data. Wang and Kennedy introduced a novel algorithm for letter-to-number conversion designed to optimize PCA [17]. In contrast, Wang and Jiang used this frequency-based PCA analysis to track COVID-19 genome mutations in 20,000 samples [25].

This paper investigates the role of prey interaction, particularly diet preference, in the divergence of conotoxins using the bioinformatic approach. Fast Fourier transforms (FFT) and principal component analysis (PCA) were applied in MSA. It aims to reveal clustering events among peptide sequences and investigate the role of diet in conotoxin divergence. Additionally, this study aims to analyze the results at the molecular level through structural element identification. It aims to identify homologous residues among related sequences within the three diet-based subgroups (molluscivorous, vermivorous, and piscivorous) to give insights into the conservation patterns of clustering events. *A superfamily* was used as a model protein group for the molecular analyses due to the vast amount of literature that utilized nAChR receptors. 

## 2. Materials and Methods

### 2.1. Data Structure Based on PCA 

#### 2.1.1. Peptide Sequences Data Collection and Preparation 

Conotoxin peptide sequences were retrieved from the ConoServer conopeptide database (http://www.conoserver.org) accessed on 22 March 2022 in .fasta format [26,27]. Entries were prepared by removing duplicate, patented, and synthetic sequences from the dataset. Data samples included the precursor sequences. Data feature extraction and selection were based on the gene superfamily and diet type: molluscivorous, piscivorous, and vermivorous. In addition, the organism region feature was also extracted. The ‘all’ option was selected for other filter criteria. The gene superfamily options were varied. In each superfamily search, sequences were sorted and grouped based on the diet type to retain the feature of the large dataset. 

#### 2.1.2. Multiple Sequence Alignment

The sorted conotoxin sequences from each gene superfamily were fed as inputs into the MSA program. The amino sequences were aligned using the MAFFT alignment server (https://mafft.cbrc.jp/alignment/server/) accessed on March 2022 [22]. ‘Same as input’ was selected in the output option to preserve sorting. The automatic strategy option was used to adjust the progressive and iterative refinement methods as needed. For the amino acid scoring matrix, BLOSUM 62 was selected with 1.53 and 0.00 as the default gap opening penalty and offset value. The default MAFFT homolog options were set. Jalview 2.11.2.1 desktop was used to view the MSA results via link input [28]. Sequence alignments and conservation plots were retrieved. 

#### 2.1.3. Cluster Analysis through Principal Component Analysis and Neighbor-Joining Trees

Cluster analysis was conducted to reveal the data structure using the calculate tree to generate neighbor-joining tree (NJT) or calculate PCA option in Jalview. PCA was implemented using the plug-in program with BLOSUM62 as the scoring function. The sorted groups were highlighted and merged to properly visualize clustering based on diet types. An artificial dataset was generated to test the algorithm’s performance using venom from six taxa (snakes, scorpions, spiders, cone snails, sea anemones, and insects). Sixty random sequences were retrieved from UniProt, having ten entries per taxa. Conopeptide amino acid sequences were examined for the PCA. A neighbor-joining tree was constructed for superfamilies with a small sample size using the distance-based construction phylogenetics plugin in Jalview.

### 2.2. Structural Analysis of Alpha-Conotoxins Based on Diet Preference 

#### 2.2.1. Data Collection

The peptide search was limited to the alpha family using selective filters. Wild-type protein sequences were selected, removing duplicate, patented, and synthetic peptides. For sorting, we enabled organism diet type in the result list column. The entries were sorted by diet type to retain the diet feature of the sequences even when extracted in .fasta format, which is essential for grouping after the alignment. Sequences were downloaded in .fasta format and were subjected to multiple sequence alignment using MAFFT. The allow unusual symbol was enabled, and the output order was set to ‘same as input’. For the MSA strategy, automatic was set, and BLOSUM 62 was used for the scoring matrix with a gap opening penalty of 1.53 and an offset value of 0.00.

#### 2.2.2. Structural Element Identification and Analysis

The aligned sequence was analyzed for structural element identification, conservation, consensus, and physicochemical and hydrophobicity changes. Structural element identification was conducted using the multi-harmony web tool plugged into Jalview [29]. The sorted conopeptide sequences were grouped, creating three subgroups based on diet types, and fed to multi-harmony. Conservation histograms and consensus logos were calculated using Jalview. Zappo was utilized for observing changes in amino acid residues based on Zappo’s physicochemical color scheme, while hydrophobicity changes were based on Kyte and Doolittle’s method for displaying the hydropathic character of a protein [30]. 

## 3. Results

### 3.1. Database Search and Feature Extraction

A database search identified that out of 45 gene superfamilies, 27 had enough elements for further analysis. Among these, gene superfamily O2/contryphans, B1/conantokin, C/contulakin, and M/conomarphin are representatives of disulfide-poor peptides. Diet preferences varied in each gene superfamily. Table 1 summarizes the data features based on diet type and organism region through the annotations listed by ConoServer. Notably, conopeptides from the conodipine, G2, I3, K, Q, R, and Y gene superfamilies are diet-specific regardless of organism species origin (Table 2). These conopeptides, except for conodipine, are only expressed in cone snails that eat worms (vermivorous), while conodipine is only expressed in piscivorous cone snails. Moreover, some conopeptides are absent in specific diet types. They include conopeptides from the B1, D, C, divergent, and L gene superfamilies. They are not expressed in molluscivorous cone snails, while conopeptides from the P and U gene superfamilies are not expressed in piscivorous cone snails. Conopeptides from large gene superfamilies (more than 100 entries) from the A, T, M, O1, and O2 gene superfamilies are expressed in the three diet types. 

Geographical region feature extraction revealed that some conopeptides are isolated only from cone snails in specific regions. Conopeptides from C, H, insulin, J, S, I3, K, Q, Y, and U are only isolated from the Indo-Pacific region. In comparison, the conodipine and R conopeptides are isolated only in the Eastern Pacific and Western Atlantic/Caribbean regions, respectively. The bulk (more than 50%) of conopeptides are isolated in cone snails from the Indo-Pacific region. Interestingly, an exclusive relationship between molluscivorous and Pacific-region conopeptides was observed. From the database search, all conopeptides from molluscivorous snails were isolated only in the Pacific region. It is also essential to note that all conopeptides from each of the 27 gene superfamilies were isolated from different species of cone snails and not from a single species (see Table 1; no group is marked with c). 

### 3.2. Multiple Sequence Alignment

**Figure 1:**
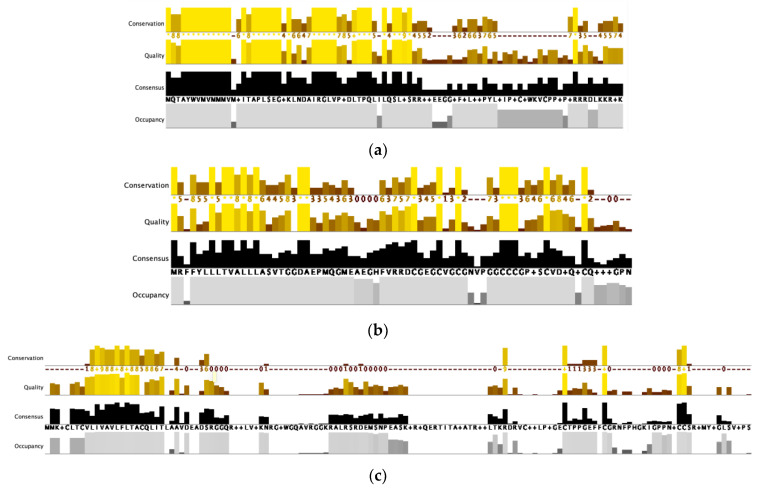
Quantitative alignment annotation visualized as histograms: (**a**) C gene superfamily; (**b**) G2 gene superfamily; and (**c**) O1 gene superfamily. Bright yellow and light gray indicate high scores.

MSA featured the three-domain organization. The conservation patterns are reminiscent of the characteristic prepropeptide organization, which is prominent in all superfamily inputs. The quantitative sequence annotation presented in the histograms (Figure 1) showed high amino acid conservation near the N-terminus, less conservation in the middle, and hypermutation with high cysteine conservation near the C-terminus. This pattern is highly prominent in gene superfamilies with small samples, such as in C, conodipine, D, divergent MSTLGMTLL, E, G2, K, L, P, Q, and R (Figure 1a,b). Nonetheless, these cases are not isolated because other groups with large samples also exhibited this pattern such as in O1, O2, T, and M (Figure 1c). No evidence of cysteine conservation was found in the disulfide-poor conopeptides, as expected, compared to the other conotoxin samples (Figure 1a). Amino acid residues were aligned based on BLOSUM62. High-quality alignment scores are prominent in the histograms, represented by a bright yellow color. In the consensus pane, a high percentage identity (PID) is prominent in all amino acids near the N-terminus and cysteine residues near the C-terminus, indicative of preserved amino acids in the signal sequence and cysteine framework across the gene superfamilies. This finding is further validated by the absence of cysteine consensus in disulfide-poor conopeptides. Occupancy or the number of aligned positions is high, especially in the small datasets.

### 3.3. Principal Component Analysis

**Figure 2:**
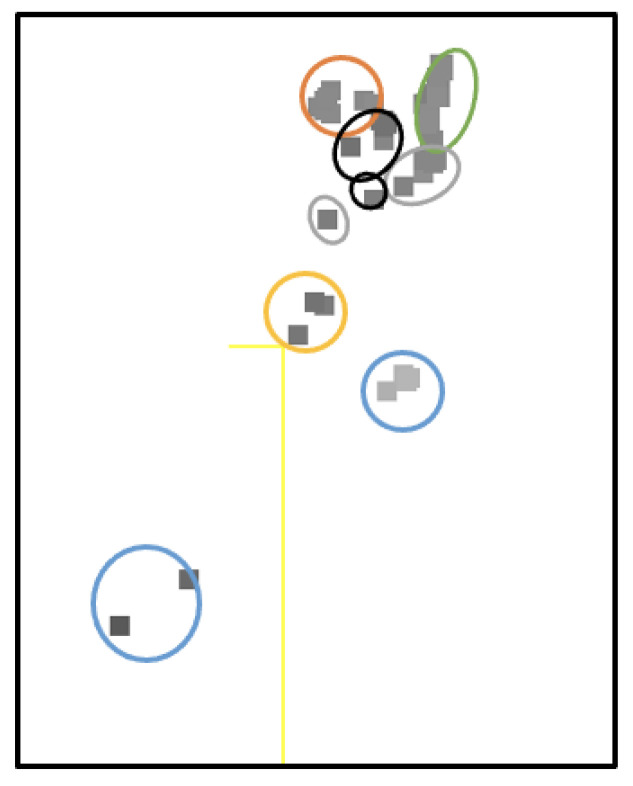
PCA plot of the artificial dataset. Scorpion (gray), conus (green), anemone (blue), spider (orange), bee (black), and snake (red).

**Figure 3:**
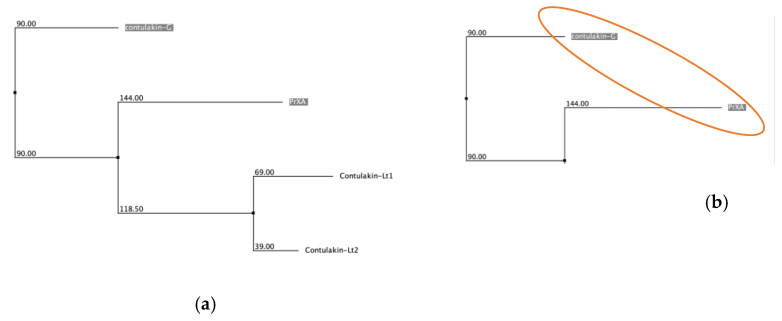

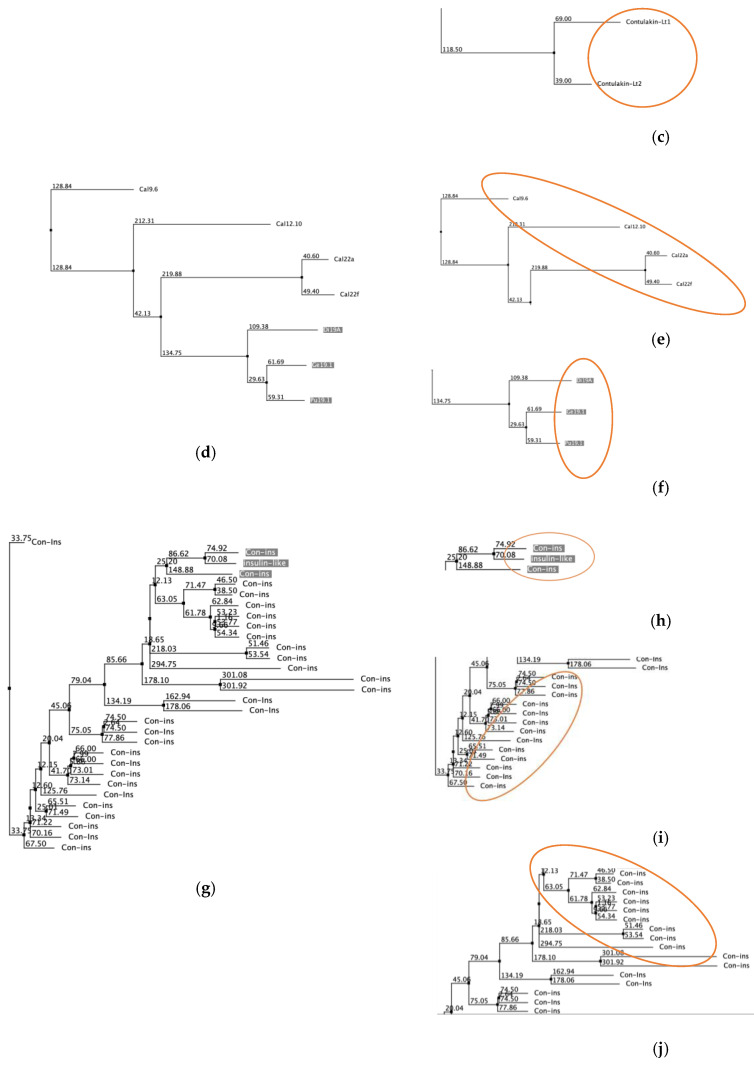
Neighbor-joining tree dendrograms. Each branch represents a single protein sequence. Sequences encircled represent the same diet types: (**a**) C superfamily (whole), (**b**) piscivorous C superfamily, (**c**) vermivorous C superfamily, (**d**) divergent-MSTLGMTLL superfamily (whole), (**e**) piscivorous divergent MSTLGMTLL, (**f**) vermivorous divergent MSTLGMTLL, (**g**) insulin superfamily, (**h**) molluscivorous insulin superfamily (whole), (**i**) piscivorous insulin superfamily, and (**j**) vermivorous insulin superfamily.

The artificial dataset showed noticeable clustering in the venom peptide components across the six taxa (Figure 2). PCA of the aligned sequences revealed that PCA is only practical when handling a large dataset as PCA plots for small samples (Table 2, C gene superfamily) do not show clustering even if the NJT (Figure 3, C gene superfamily) suggests that samples are grouped based on a high sequence similarity. 

PCA of datasets (gene superfamilies) with more than 20 samples showed evidence of clustering events based on diet types (Table 2). Interestingly, conopeptides from vermivorous cone snails showed prominent sequence similarities, as indicated by component clusters in the PCA plots away from other diet-type clusters. These features are mainly observed in the A, I2, M, O1, O2, O3, and T gene superfamilies (Table A1). B1, on the other hand, showed prominent clustering in conopeptides from piscivorous cone snails (Table 2). In addition, the relative abundance of conopeptide isolates in each plot showed a positive selection for a specific diet type. Conopeptides from the A, I2, M, O1, O2, and T gene superfamilies are mainly from vermivorous cone snails, as indicated by the large area occupancies of components from the vermivorous column. For gene superfamilies with a small sample size, NJTs were employed. The NJT dendrograms revealed close-distance groupings for conopeptides belonging to the same diet (3). In some cases, monophyly was observed (Figure 3c, vermivorous and insulin, molluscivorous). 

### 3.4. Multiple Sequence Alignment of Alpha-Conotoxin Samples

The database search with filters resulted in 60 peptide sequences summarized and subjected to MSA. Peptides EIIB and PIC were removed from the set in the refinement process due to the unusual residue *O* from posttranslational modification. The features of these samples are based on the annotation and curation listed by ConoServer from the Conus Biodiversity website (http://biology.burke.washington.edu/conus/ accessed on 22 March 2022) and peer-reviewed literature [26,27].

**Figure 4:**
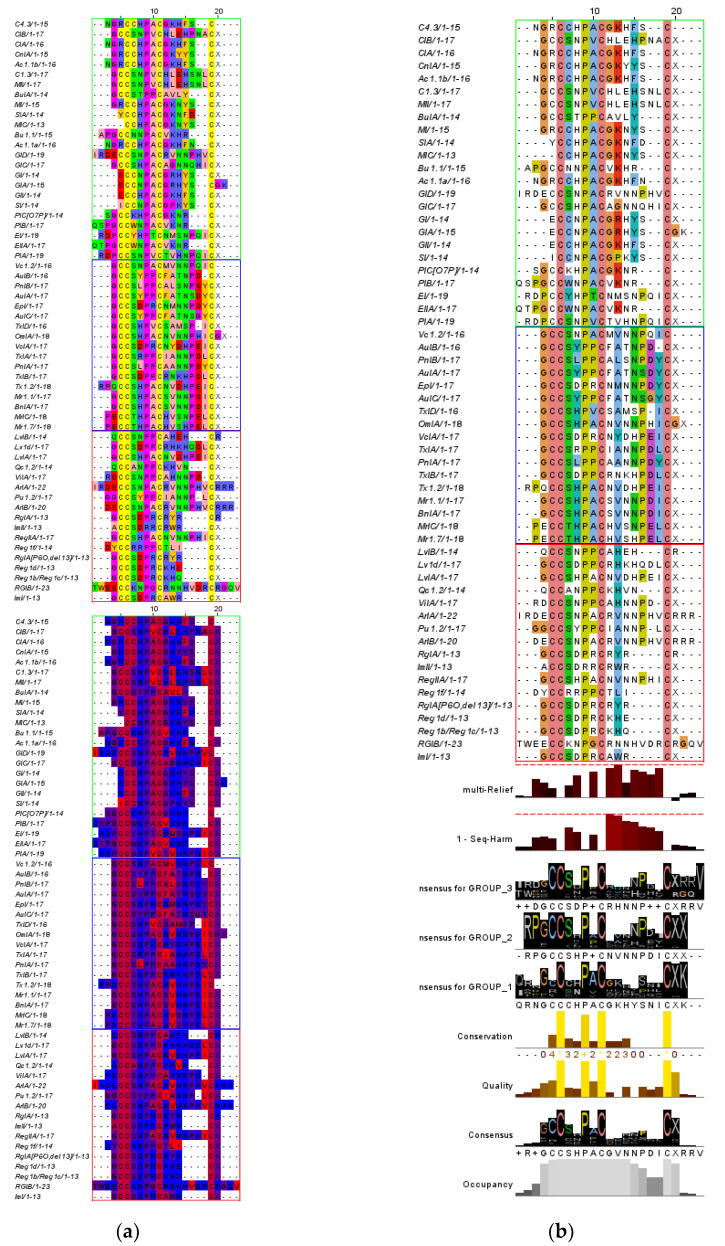
Multiple sequence alignment of the alpha-conotoxins: (**a**) CLUSTALX with histograms, (**b**) Zappo physicochemical color scheme, and (**c**) hydrophobicity color scheme. Group 1 (piscivorous) is enclosed in neon green, group 2 (molluscivorous) is enclosed in blue, and group 3 (vermivorous) is enclosed in a pink square.

The aligned sequences showed prominence of the α-conotoxin type I cysteine framework with the distinct cysteine conservation pattern of –CC–C–C– [31,32]. It was emphasized by the yellow/pink highlighting in the aligned sequences, bright yellow bars in the histograms, and the large letter C in the consensus logo (Figure 4). The prominence of the cysteine framework followed the type I pattern with a percent identity of 79%, 100%, 100%, and 55%, respectively. Between the second and third cysteine, a highly conserved proline residue was also present with a percentage identity of 98%. The consensus logo also agrees with this conservation, as indicated by the large logos for the C and P residues. The quality of these alignments was scored as 203–207, which is considered a high alignment quality as indicated by the tall bright-yellow bars on the histogram [28]. 

### 3.5. Structural Element Analysis

Sequence alignment showed the prominence of the type I cysteine framework (Figure 4). However, a homology pattern is prominent within the diet-based subgroups. In group 1 (piscivorous), there are shorter lengths, indicated by gaps and misalignments. The poor alignment of the first and second cysteine with the other subgroups indicates possible gaps due to the shorter lengths of the intervening amino acids between the first/second and third cysteine residue. In group 2 (molluscivorous), the peptides are longer with full occupancy and no gaps. In group 3 (vermivorous), there are shorter amino acids between the third and fourth amino acids.

Multi-harmony was used to determine sub-type-specific sites in the peptides, analyzing the conserved residues within diet groups but divergent from the others. Based on the multi-harmony results, positions 12, 13, 14, 15, 16, 7, 17, and 18 have the highest sequence harmony (SH) scores, while positions 16, 12, 13, 15, and 7 have the highest z-scores (Table 3). These parameters measure the degree of overlapping and sequence reliability. A 0 SH score indicates nonoverlapping residues for each subgroup; hence, the lower the score, the better. The more negative the z-score, the higher the empirical significance and the higher the reliability. Furthermore, positions 12, 13, 15, 16, 18, and 10 have the highest multi-relief weight, indicating conservation within groups. With these three parameters, positions 12, 13, 15, and 16 are the identified specificity residues that group the peptide sequences. These results are summarized as histograms in Figure 4.

The amino acid character was analyzed for these four positions (Figure 4 and Figure 1a). In the 12th position, group 1 has a higher glycine conservation than the other subgroups. In the 13th position, group 1 has high positively charged amino acids, and groups 2 and 3 have high hydrophobic/aliphatic amino acids. In the 15th position, group 2 has a high hydrophilic arginine occupancy, while group 1 has high tyrosine and phenylalanine aromatic amino acids. In the 16th position, group 1 has high hydrophilic serine substitution, group 2 has high proline substitution, and group 3 has prominent gaps. Table 4 summarizes the amino acid characters per position and their percentages based on the consensus logo (Figure 4). 

## 4. Discussion

### 4.1. Principal Component Analysis

Multiple sequence alignment was used to develop and test hypotheses based on protein structure, function, and phylogenies. High-quality alignments from large datasets are challenging to generate and interpret due to risks of misalignments, computational burden, and the multivariate nature of peptide sequences [18,20]. In this study, FFT and PCA were explored to help with these problems and to ultimately investigate conotoxin divergence patterns based on peptide sequences available in ConoServer.

Feature extraction and sorting revealed that conopeptides in each gene superfamily are isolated from the main three diet types based on the prey they subdue. They can be classified as isolates from piscivorous species that immobilize and gulp fish, molluscivorous species that feed on other gastropods, and vermivorous species that feed on polychaete worms [16]. Generalists are available in ConoServer but were omitted to increase the specificity of the pattern analysis. Strikingly, several gene superfamilies are diet-specific, meaning that these groups only contain peptides that target one diet type. It was found that peptides from the G2, I3, K, Q, Y, and R gene superfamilies are only expressed in vermivorous cone snails, while conodipine is expressed only in piscivorous cone snails (Table 1). Our findings are consistent with previous reports where several novel conotoxins were isolated only in vermivorous and piscivorous species [33,34]. 

To assess superfamilies that house conopeptides targeting more than one diet type, NJTs and PCA distinguished peptide sequence similarities based on diet preference using the BLOSUM62 scoring matrix. NJTs were used for calculating and visualizing gene superfamilies with a small number of samples because PCA failed to display the appropriate similarity space or proximity, as similar sequences tend to lie far from each other in space, such as in the case of the C superfamily (Table 2) which shows similarity in the NJT dendrogram (Figure 3). It appeared that distances between points of similar sequences were enhanced due to the absence of other components. The NJT dendrograms revealed that peptide sequences within the same diet type have nearer distances than sequences from others (see divergent MSTLGMTLL and insulin in Figure 3). In some cases, sequences within the same diet type are located in the same node (see C, vermivorous; insulin, molluscivorous in Figure 3), implying divergence events based on diet type. Both patterns suggest sequence similarities in peptides isolated from cone snails with the same diet. 

Principal component analysis was used to examine the data structure of the complex peptide sequences within superfamilies to investigate the role of diet preference in conotoxin divergence. PCA revealed prominent clustering in the similarity spaces (Table 2). The components, which are data features of the peptide sequences, were generated by an eigenvector decomposition formed from the sum of the BLOSUM62 scores at the aligned position between each pair of sequences. In this study, the score matrix size can be huge, ranging up to a 100 × 100 matrix and more for large gene superfamilies. Interpreting a large data matrix can be tedious. Hence, to maintain objectivity, PCA was used to reduce dimensions to find directions of differences and visualize them by using the axes [20]. The PCA algorithm was tested using an artificial dataset composed of highly similar venom peptide sequences from six different taxa. The PCA plots showed positive clustering per taxa as expected (Figure 2). It suggests that the algorithm [23,28] satisfies objectivity and reproducibility [20]. PCA of large gene superfamilies exhibited positive clustering based on diet types. The findings imply there is little variation in conopeptide sequences from the same diet group, as evidenced by the PCA plots for each gene superfamily (Table 2). It is noteworthy that the peptide sequences in each gene superfamily originated from different species of cone snails, meaning the vermivorous peptide samples in the A superfamily (Table 2) originated from multiple species. Hence, peptide sequence similarity bias based on where they are expressed can be neglected.

**Figure A2:**
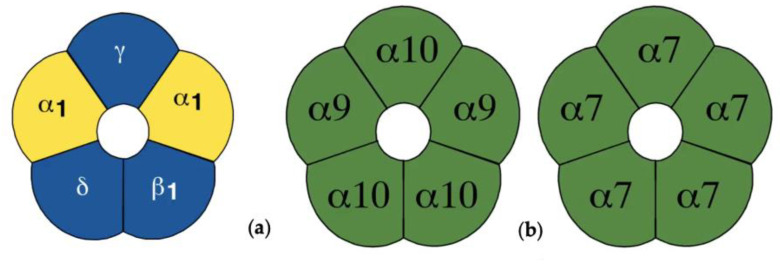
Different nAChRs found in muscle synapse: (**a**)mostly in vertebrate and (**b**) mostly in invertebrate. Source: adapted from [35].

Sequence similarity based on diet type has far-reaching implications as it explores the role of diet preference on the evolutionary divergence of conotoxins. Diet has always been thought to be a significant driving force shaping venom composition and evolutionary patterns because venom is highly connected to a species’ ability to apprehend prey [13]. Inside prey are target receptors where conotoxins bind and act to reach specific physiological endpoints [1]. PCA revealed data structures and patterns on peptide sequences grouped in gene superfamilies. It is important to note that the gene superfamily classification is based on signal sequence and cysteine framework sequence similarities, and subsets to it are pharmacological families that have a functional role in classifying conotoxins based on the specific target receptor where they bind [32]. Hence, structure-function-wise, peptide sequences in each gene superfamily should be expected to have significant similarities. Interestingly, the data structure within gene superfamilies showed peptide sequences similarities based on the cone snail diet preference. These findings may imply that conotoxins diverged accordingly to increase affinity and fit target receptors found in polychaete worms, fish, or mollusks. Furthermore, hypermutation of the M-regions presumably fine-tunes conotoxins to increase their binding affinity in their specific prey receptors. There is evidence of the structural difference between nicotinic acetylcholine receptors (nAChRs) in invertebrates (e.g., nematodes and polychaetes) and vertebrates (Fig.A2). In fire worm-eating cone snails (vermivorous), the venom contains many peptides specific to the homopentamer homolog of the nAChR that only have alpha subunits (Fig.A2). It was presumed that the abundance of very specific conotoxins in worm-hunting cone snails serves their purpose to act on the all-alpha subunit nicotinic receptor found in the neuromuscular junction of fire worms [35]. 

Conotoxin divergence based on diet can be beneficial for receptors binding to their prey. Due to environmental fluctuations (e.g., sudden climate changes or catastrophes), rapidly changing prey, predators, and competition force conotoxins to adapt rapidly by diverging, as if it is a mechanistic way to evolutionarily succeed by really diversifying [1,36]. In conotoxins, venom component diversity plays a crucial role in receptor binding to reach the desired physiological endpoints. Olivera described this as the combinatorial library search strategy, an optimum evolutionary technique of cone snails to evolve new peptides in their venom to generate neuropharmacological diversity [1]. Early accounts revealed that multiple conotoxins play a role in producing the desired physiological response in prey capture, as individual venom components did not produce the same effect as crude venom. It tells us that conopeptides work together in groups (or cabals) to reach a physiological endpoint [1,37]. Hence, increased spontaneous mutation rates in the M-regions of conotoxins are beneficial because they enrich the combinatorial pool of conopeptides to achieve the appropriate formulation to adapt to a particular hunting situation [6,38]. Tt appears that cone snails evolved to fine-tune their conopeptide armory to respond to evolutionary pressures by producing powerful cabals of peptides selective for the cone snail’s prey of choice [3]. 

Lastly, several unusual patterns emerged from the data. Most peptides in the database are isolated from the Indo-Pacific region. Strikingly, molluscivorous snails are exclusive to cone snails located in the Pacific region (Table 1). This result implies that venom variation is affected by environmental conditions. The role of climate, seasonal changes, and temperature show positive changes in venom variation in scorpions and snakes [39,40]. Future studies on this topic can be pursued to establish the role of the geographical distribution in cone snail venom composition and diversity. 

### 4.2. Structural Element Analysis

Prey shifts can accelerate conotoxin diversity. Due to environmental fluctuations, cone snails must adapt to rapidly changing predators, prey, and competition. Food resource utilization is among the critical evolutionary events that can lead to biodiversity on Earth, and these shifts open opportunities for studying the underlying molecular changes [41]. Morphological observations and sequencing efforts indicate a vermivorous ancestry that evolved into molluscivorous and piscivorous diets. These evolutionary reconstructions based on curated databases show that ancestral cone snails preyed on marine worms and evolved the capacity to prey on mollusks and fish [26,27]. These findings align with the molecular phylogeny analysis of Puillandre et al. and Aman et al., suggesting two separate events in the Miocene era which triggered the generation of fish-hunting and mollusk-hunting cone snail lineages [42,43]. These prey shifts led to a series of adaptive radiations that continued to present, as evidenced by fossil records showing few fish-hunting and mollusk-hunting cone snail species in the geologic past, anatomical specialization, and now, the increasing role of venom specialization [44,45]. 

All cone snails use their venom as the primary weapon for prey capture. In our previous data mining study [7], sequences in each superfamily clustered based on diet types, meaning that conopeptides from mollusk-hunting or molluscivorous cone snails have greater sequence similarities than conopeptides belonging to other groups. These findings opened opportunities for subgroup identification based on diet types that may play functional roles in conotoxin affinity to target receptors. 

**Figure A3:**
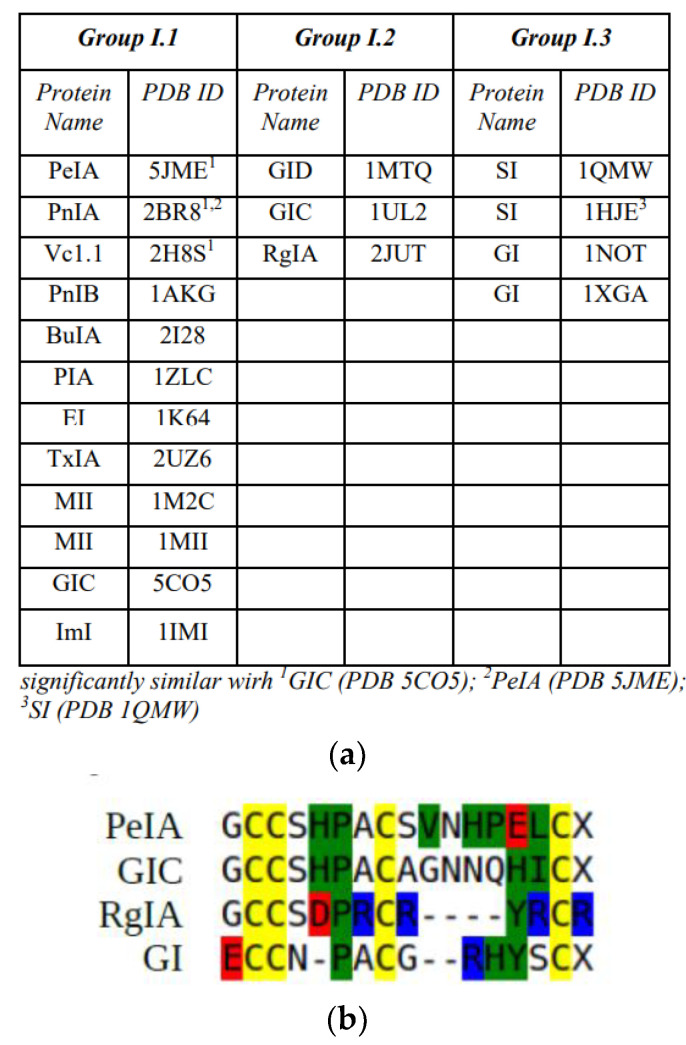
Three subgroups in the type I cysteine framework: (**a**) table summary of the three subgroups with their PDB IDs; (**b**) sequence alignment of the amino acid residues representative of the three subgroups. Adapted from [11].

Molecular analyses revealed an interesting pattern when sequences from the α-conotoxin pharmacological family were aligned and analyzed. All samples showed the type I cysteine framework (-CC--C--C--) that is known to potently and selectively target nAChR subtypes [32,46]. However, within the alpha-conotoxin family, subgroups were apparent based on structure similarity. Conopeptides from fish-hunting or piscivorous cone snails have a smaller number of intervening amino acids between the first/second and third cysteine residue, indicated by the misalignment of these positions (Figure 4). Furthermore, conopeptides from worm-hunting or vermivorous cone snails showed shorter intervening amino acids between the third and the fourth cysteine residue, as indicated by the gaps near the C-terminus. In contrast, molluscivorous cone snails have full occupancy between cysteine residues (Figure 2). These results reveal that the number of intervening amino acids between cysteines may play a functional role in conopeptides, which are unexpectedly based on diet subgroups. The most striking result from the data is that these classifications were observed before in the structural determinant study conducted by Gomez et al. but they did not show what caused the subgrouping [11]. Their analysis revealed that type I alpha-conotoxins fall into at least three distinct categories (as in Figure A3). Peptides from different subgroups showed dissimilar α-conotoxin backbones, indicated by gaps in their structural alignment. Our results share similarities with their findings; however, we identified that these subgroups are based on cone snail diet types. Notice that in Figure A3, group 1.1 contains a sample from the molluscivorous cone snails such as TxIA from mollusk-hunting *C. textile*, group 1.2 contains samples from vermivorous cone snails such as RgIA from worm-hunting *C. regius*, and group 1.3 are conopeptides from piscivorous cone snails such as GI from fish-hunting *C. geographus.* These three subgroups are classified based on the diet preference where they take action, indicating possible insertions and deletions between cysteine residues that caused venom specificity. 

Structural element analysis revealed positions containing specificity residues located between the third and fourth cysteine in a type I cysteine framework (Table 3). The tests identified that positions 12, 13, 15, and 16 contain structural elements that are nonoverlapping and statistically reliable based on their high SH score and z-score (Table 3). Furthermore, their high multi-relief weights indicate that these positions are conserved within subgroups but divergent between other groups. These findings may have identified structural elements critical in function specificity, providing insights into why these conopeptides group and act on specific diet types. Overall, it was found that α-conotoxins have structural homology within the same diet subgroup but are divergent from other diet subgroups.

Overall, strong conservation patterns are prominent in molluscivorous peptides. The tests revealed highly conserved hydrophobic, hydrophilic, and small residues intervening the third and fourth cysteine. Similarly, the prominence of gaps showed shorter intervening amino acids between cysteine residues for piscivorous and vermivorous peptides. 

## 5. Conclusions

In summary, this paper used PCA and structural element analysis to investigate the role of diet preference in the evolutionary divergence of conotoxins. Multiple sequence alignment using FFT was able to align a large number of sequences rapidly with a high alignment quality, conservation reminiscent of the prepropeptide organization, and a high occupancy. NJTs and PCA indicated peptide sequence similarities in conotoxins isolated in cone snails with the same diet preference. Our findings suggests that certain groups of conopeptides are dominant or exclusive in specific diet types. Taken together, these results suggest that conotoxin divergence does not happen randomly but perhaps through multiple intermediate steps through conservation and hypermutation events to increase selectivity and potency towards the receptors of their prey preferences, overall contributing to the success of cone snails as a predatory species. We also presented a molecular analysis to present the molecular events that led to peptide similarity based on diet preference. Homologous regions and conserved residues within groups but divergent between other groups were identified. Notably, in the type I framework, homologous regions are located between the third and fourth cysteine residues, which were believed to play a significant role in diet-specific venom specialization. A hydrophobic, hydrophilic, and small amino acid character showed high conservation in the molluscivorous conopeptides. Piscivorous and vermivorous conopeptides showed shorter intervening amino acids between the first/second and third and third and fourth cysteine residues, respectively. The evidence from this study suggests that the character, the length of the intervening amino acids between the cysteine residues, and the polypeptide length contributed to diet subgrouping. Taken together, this study implies that conotoxin divergence co-occurs with the changes in prey receptors. Prey shift events may have accelerated structural changes in conopeptides for successful food acquisition, resulting in venom specialization evidenced by sequence similarity based on diet types. Additional molecular analyses are needed to consider the target receptors’ molecular evolution to establish the mechanism and effects of prey receptor mutations on conotoxins. 

## Figures and Tables

**Table 1:** Gene superfamily features.

Gene Superfamily	Data Feature Annotation	
	Diet Type ^a^	Organism Region ^b^
A, T	M, P, V	IP, WAC, EP, EAM
B1, D	P, V	IP, WAC, EAM
C	P, V	IP
Conodipine	P	EP
Divergent MLLTVA and MSTLGMTLL, L	P, V	EP, IP
E	M, P, V	IP, WAC, EAM
G2	V	IP, EP
H, Insulin, J, S	M, P, V	IP
I1	M, P, V	IP, EP
I2	M, P, V	IP, WAC
I3, K, Q, Y	V	IP
M, O1, O2	M, P, V	IP, EP, EAM
O3	M, P, V	IP, EAM
P	M, V	IP, EP
R	V	WAC
U	M, V	IP

^a^ Molluscivorous (M), piscivorous (P), and vermivorous (V). ^b^ Eastern Atlantic and Mediterranean (EAM), Eastern Pacific (EP), Indo-Pacific (IP), South Africa (SA), and Western Atlantic and Caribbean (WAC).

**Table 2:** Principal component analysis plots.

Gene Superfamily	PCA Plots ^a^			
	Overlay	Molluscivorous	Piscivorous	Vermivorous
A	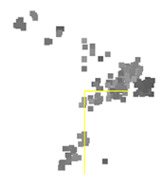	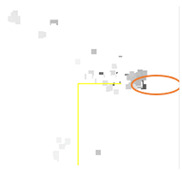	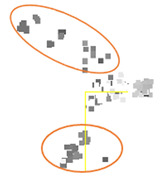	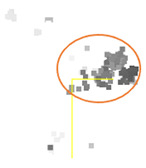
B1	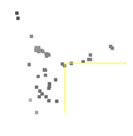		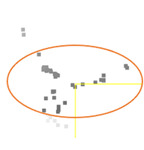	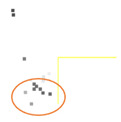
C	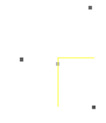		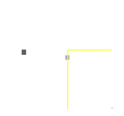	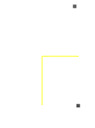
O1	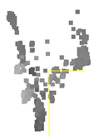	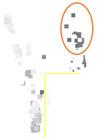	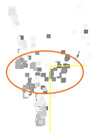	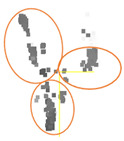
T	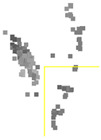	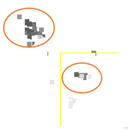	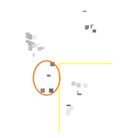	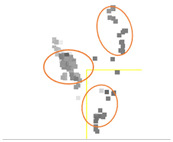

^a^ Each point represents a single protein sequence. Darker colors represent sequence similarities (white to gray to black colors). Red circles represent observed clustering events. Yellow lines represent the axis scale.

**Table 3:** Multi-harmony structural element analysis scores.

Alignment	Sequence Harmony		Multi-Relief
	Score	Z-score	Weight
12	0.42	−7.01	0.41
13	0.44	−7.80	0.45
14	0.53	−5.34	0.20
15	0.54	−9.04	0.38
16	0.62	−12.94	0.36
7	0.63	−8.74	0.17
17	0.63	−6.66	0.32
18	0.69	−5.69	0.41
8	0.71	−4.87	0.30
10	0.75	−5.73	0.36
5	0.80	−4.37	0.13
12	0.42	−7.01	0.41
13	0.44	−7.80	0.45
14	0.53	−5.34	0.20

Alignment

Sequence Harmony

Multi-Relief

**Table 4:** Specificity residue identification summary.

Position	Subgroup	Amino Acid	Property	Percentage (%)
12	I	Glycine	Small/conformationally special	54
	II	Low consensus	–	–
	III	Arginine, Lysine	Positively charged/basic	59
13	I	Lysine, Arginine	Positively charged	62
	II	Valine, Alanine, Leucine, Methionine	Aliphatic/hydrophobic	88
	III	Low consensus	–	–
15	I	Tyrosine, Phenylalanine	Aromatic	54
	II	Asparagine, Histidine	Hydrophilic, positively charged	95
	III	Gap prominence	–	–
16	I	Serine	Hydrophilic	63
	II	Proline	Small/conformationally special	88
	III	Gap prominence	–	–

**Figure A1:**
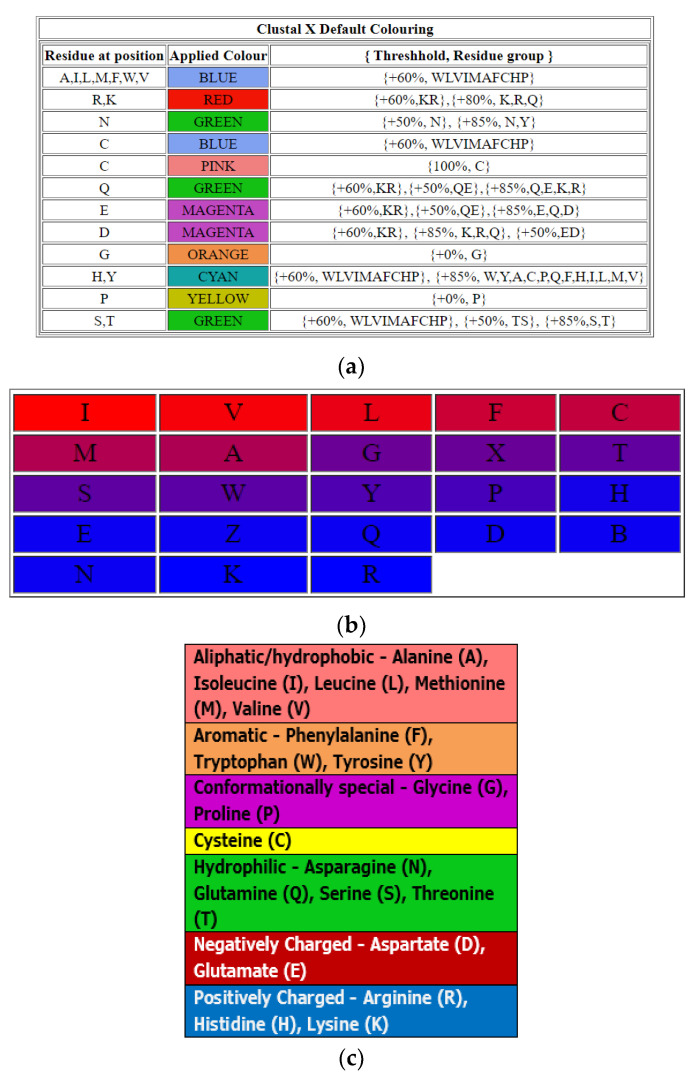
Alignment colorscheme: (**a**) CLUSTALX based on the threshold criteria and amino acid profile; (**b**) hydropathic character with the most hydrophobic residue being red, and blue being the most hydrophilic; (**c**) physicochemical character. Adapted from the documentation of Jalview [28].

## Data Availability

Publicly available datasets were analyzed in this study. This data can be found here: [https://www.conoserver.org/, accessed on 22 March 2022].

## Appendix A

**Table A1:** Principal component analysis plots.

Gene Superfamily	PCA Plots			
	Overlay	Molluscivorous	Piscivorous	Vermivorous
A				
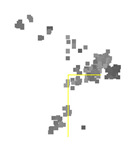
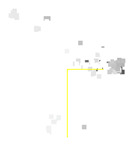
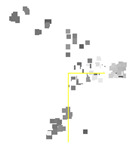
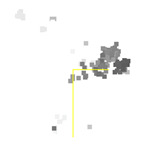
B1				
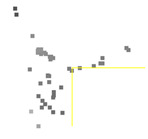
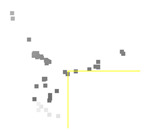
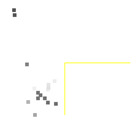
C				
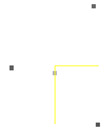
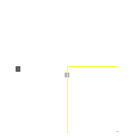
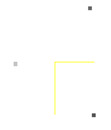
B1				
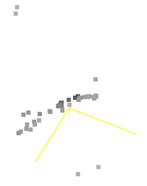
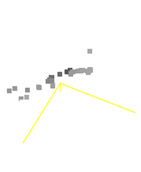
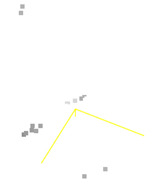
H				
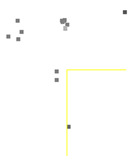
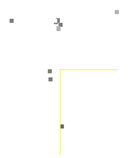
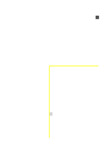
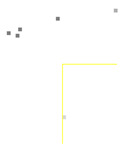
Insulin				
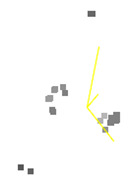
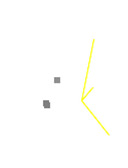
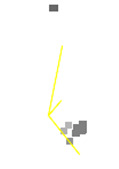
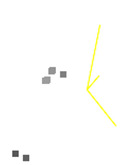
I1				
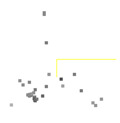
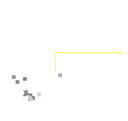
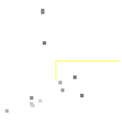
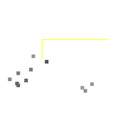
J				
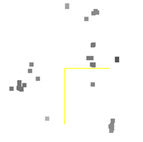
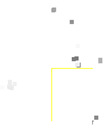
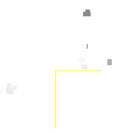
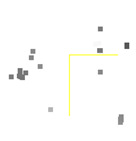
M				
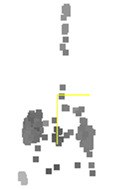
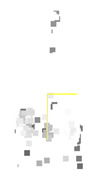
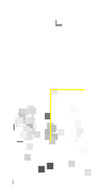
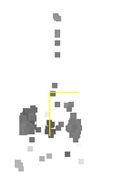
O1				
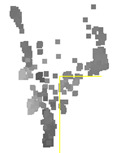
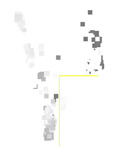
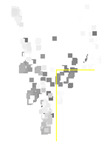
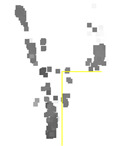
O2				
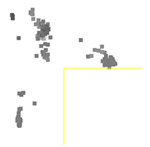
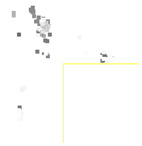
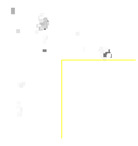
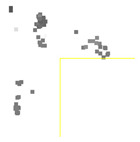
O3				
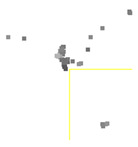
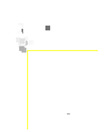
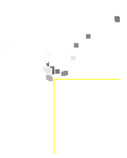
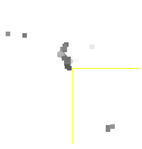
T				
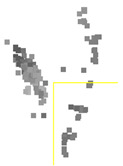
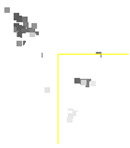
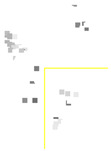
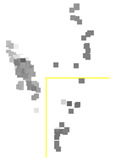

**Table A2:** Neighbor-joining trees for small sample sizes.

Gene Superfamily	NJT Dendrograms		
	Molluscivorous	Piscivorous	Vermivorous
C			
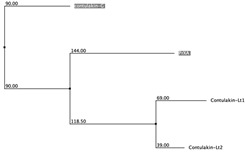
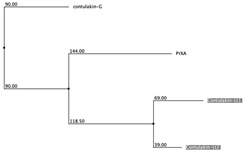
Divergent M—L-LTVA			
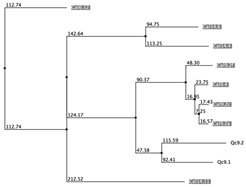
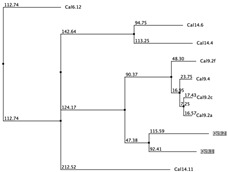
Divergent MSTLGMTLL-			
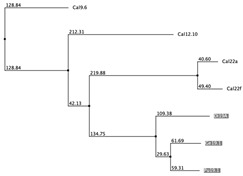
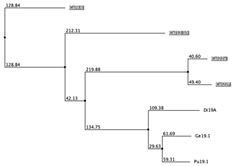
H			
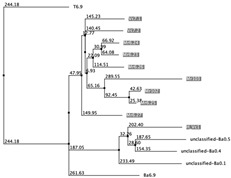
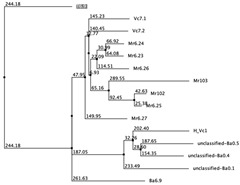
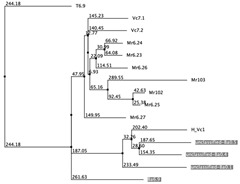
Insulin			
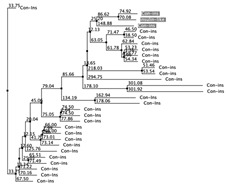
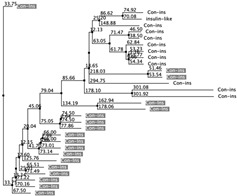
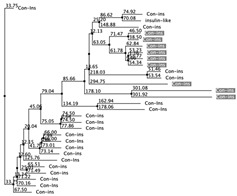
L			
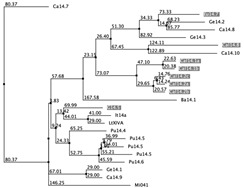
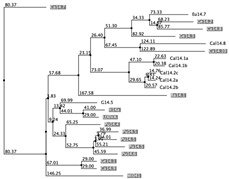
P			
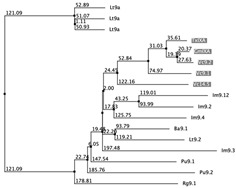
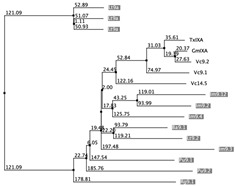
S			
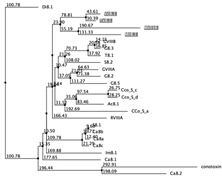
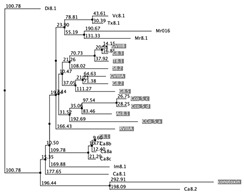
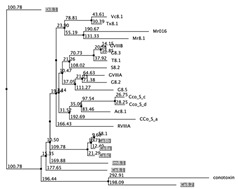
U			
